# Functional Studies of Sex Pheromone Receptors in Asian Corn Borer *Ostrinia furnacalis*

**DOI:** 10.3389/fphys.2018.00591

**Published:** 2018-05-23

**Authors:** Wei Liu, Xing-chuan Jiang, Song Cao, Bin Yang, Gui-rong Wang

**Affiliations:** ^1^State Key Laboratory for Biology of Plant Diseases and Insect Pests, Institute of Plant Protection, Chinese Academy of Agricultural Sciences, Beijing, China; ^2^School of Plant Protection, Anhui Agricultural University, Hefei, China

**Keywords:** odorant receptors, ligands, single sensillum recordings, olfactory, *Xenopus* oocytes

## Abstract

Lepidopteran insects use sex pheromones for sexual communication. Pheromone receptors expressed on peripheral olfactory receptor neurons (ORNs) are critical part to detect the sex pheromones. In genus *Ostrinia*, several pheromone receptors were functional analyzed in *O. nubilalis* and *O. scapulalis* but the knowledge in *O. furnacalis* was rare. In this study, seven pheromone receptors were deorphanized by heterologous expression system of *Xenopus* oocytes. Functional types of *sensilla trichoidea* were classified by single sensillum recordings to interpret the response pattern of olfactory sensory neurons to *Ostrinia* pheromone components. OfurOR4 and OfurOR6 responded to the major sex pheromone Z/E12-14:OAc. OfurOR4 is the main receptor for both Z/E12-14:OAc and OfurOR6 mainly responded to E12-14:OAc. Functional differentiation of gene duplication were found between OfurOR5a and OfurOR5b. OfurOR5b showed a broad response to most of the pheromone components in *O. furnacalis*, whereas OfurOR5a was found without ligands. OfurOR7 showed a specific response to Z9-14:OAc and OfurOR8 mainly responded to Z11-14:OAc and E11-14:OAc. OfurOR3 did not respond to any pheromone components. Our results improved the current knowledge of pheromone reception in *Ostrinia* species which may contribute to speciation.

## Introduction

Sex pheromone has been used by organisms for sexual communication, this remarkable trait is representative in insects especially for Lepidopterans (Symond et al., 2011). Male could detect and respond to female pheromone over long distance, e.g., 11 km for emperor moth *Pavonia pavonia* (Regnier and Law, 1968). Moth percept the sex pheromone via the pheromone sensitive *trichoid* sensilla distributed on their antennae. The entire olfactory system is heavily dependent on the types of receptors expressed in peripheral olfactory receptor neurons (ORNs; also called olfactory sensory neurons) which housed in the olfactory sensilla (Leal, 2013). This has been proved unambiguously by expressing an allospecific pheromone receptor *PxylOR1* from the diamondback moth in the ORN that houses the bombykol receptor BmorOR1 in the silkworm moth, *Bombyx mori.* Electrophysiological and behavioral experiments showed that PxylOR1-expressing male silkworm moths responded equally to bombykol (E10Z12-16:OH) and Z11-16:Ald (Sakurai et al., 2011).

The genus *Ostrinia* (Lepidoptera: Crambidae) consists of 21 species worldwide and served as the model system for research of pheromone communication. Several species in this genus are important agricultural pests such as *O. nubilalis* and *O. furnacalis* (Mutuura and Munroe, 1970; Ohno, 2003). The species in this genus use relatively simple components (Z9-14:OAc, E11-14:OAc, Z11-14:OAc, E12-14:OAc, Z12-14:OAc and E11-14:OH) for the recognition among individuals (Roelofs et al., 1985; Huang et al., 1998a,b,c; Ishikawa et al., 1999a,b; Takanashi et al., 2000). Eight pheromone receptors and odorant receptor co-receptor have been successfully functionally characterized either *in vivo* or *in vitro* among *O. furnacalis, O. nubilalis, O scapulalis*, and *O. latipennis* (Miura et al., 2009, 2010; Wanner et al., 2010; Leary et al., 2012; Yang et al., 2016).

Asian corn borer, *O. furnacalis*, is a grievous pest in China and causing serious damage on economic crop maize for 10–30% yield lost (Wang et al., 2000). In addition, this species fed on various host (over 27 species) belonging to nine families (Yuan et al., 2015). Females of *O. furnacalis* use Z12-14:OAc and E12-14:OAc with the ratio of 1:1 as their major sex pheromone components to attract males (Cheng et al., 1981; Huang et al., 1998b). Although the pheromone receptors were functionally characterized in the sibling species such as *O. nubilalis* and *O scapulalis*, only one pheromone receptor (OfurOR4) and an odorant receptor co-receptor (OfurOR2) has been deorphanized in *O. furnacalis* (Leary et al., 2012; Yang et al., 2015, 2016; Zhang et al., 2015). The functional types of the sensilla have been described by Takanashi et al. (2006) and Domingue et al. (2007). In this study, all the pheromone receptors in *O. furnacalis* were functionally characterized using *Xenopus* oocytes system. In addition, single sensillum recordings were carried out to confirm the ORNs response for detecting the pheromones.

## Materials and Methods

### Insects

*Ostrinia furnacalis* was maintained under laboratory conditions with artificial diet at 28°C, 14:10 (L:D), 60% relative humidity. Pupae were placed in tube individually for eclosion. Two-day-old adults were used in the present study. Male antennae were removed and frozen in liquid nitrogen immediately, then stored under -80°C until use.

### Pheromone Components

The pheromone components including (Z)-9-tetradecenyl acetate (Z9-14:OAc), (Z)-11-tetradecenyl acetate (Z11-14:OAc), (E)-11-tetradecenyl acetate (E11-14:OAc), (E)-11-tetradecen-1-ol (E11-14:OH), (Z)-12-tetradecenyl acetate (Z12-14:OAc), E-12-tetradecenyl acetate (E12-14:OAc) (95% minimum purity) were purchased from Nimrod Inc. (Changzhou, China). For *Xenopus* oocyte system, chemicals were prepared in dimethyl sulfoxide (DMSO) to form the stock solutions (1 M) and stored at -20°C. The stock solution was diluted in 1× Ringer’s buffer (96 mM NaCl, 2 mM KCl, 5 mM MgCl_2_, 0.8 mM CaCl_2_, and 5 mM HEPES pH 7.6) before experiments. 1× Ringer’s buffer was used as a negative control. For single sensillum recording, each pheromone compound was prepared as 1 μg/μl in hexane solution and stored at -20°C. The hexane was used as a negative control.

### RNA Extraction and cDNA Synthesis

Total RNA was isolated from male antennae with TriZol reagent (Invitrogen, Carlsbad, CA, United States) according to the manufacturer’s instruction. The cDNA was synthesized using RevertAid First Strand cDNA Synthesis Kit (Thermo Scientific) after a DNase I (Thermo Scientific) treatment. The quality of RNA was verified by Nanodrop ND-1000 spectrophotometer (NanoDrop Products, Wilmington, DE, United States) and gel electrophoresis.

### Cloning of Pheromone Receptors in *O. furnacalis*

Full length of ORF encoding odorant receptors of *O. furnacalis* was obtained from antennal transcriptomic analysis and amplified by PCR using primeSTAR HS DNA polymerase following the manual (Takara, Dalian, China) (Yang et al., 2015). Primers used in this study were listed in **Supplementary Table S1**. Transmembrane domains were predicted by TMHMM Server Version 2.0^1^ and multiple sequence alignment and identity calculation were done by the DNAMAN 6.0 (Lynnon Biosoft, United States).

### Electrophysiological Recordings Using *Xenopus* Oocyte System

Each receptor was first cloned into a blunt-vector (TransGen Biotech, China), subsequently subcloned into a PT_7_TS vector, and then took for cRNA synthesis using mMESSAGE mMACHINE^TM^ T7 Kit (Thermo Fisher Scientific). Mature healthy *Xenopus* oocytes (stage V-VII) were prepared according the description from Liu et al. (2013). Briefly, the oocytes were separated and then treated with 2 mg/ml collagenase I in washing buffer (96 mM NaCl, 2 mM KCl, 5 mM MgCl_2_ and 5 mM HEPES, pH 7.6) for 1–2 h at room temperature. The 1:1 mixture of pheromone receptor and *OfurOrco* (*OfurOR2*) cRNA (27.6 ng each) were microinjected into the oocytes. After an incubation for 4–7 days at 18°C in incubation medium (1× Ringer’s buffer, 5% dialysed horse serum, 50 mg/ml tetracycline, 100 mg/ml streptomycin, and 550 mg/ml sodium pyruvate), oocytes were recorded with a two-electrode voltage clamp. Currents induced by pheromone components (100 μM) were recorded using an OC-725C oocyte clamp (Warner Instruments, Hamden, CT, United States) at a holding potential of -80 mV. The data were acquired and analyzed with Digidata 1440A and pCLAMP 10.0 software (Axon Instruments Inc., Union City, CA, United States).

### Single Sensillum Recordings

*Sensilla trichoidea* from 2-day-old male adults were used for the recordings. Individuals were restrained in a remodeled 1 ml plastic pipette tip with an exposed head fixed by dental wax, and antenna from one side was attached to a coverslip with double-face tape. Two tungsten wire electrodes were used with one inserting into an compound eye and another into the sensilla. Ten individuals were recorded at basal (4), middle (3), and proximal (3) part of the antennae and ten sensilla were recorded for each individuals. Ten micrograms pheromone components (dissolved in hexane) were performed for each trial. Air flow was set at 1.4 L/min with a 300 ms stimulus air pulse controlled by Syntech Stimulus controller (CS-55, Syntech, Kirchzarten, Germany). AC signals were recorded (10 s, starting 1 s before stimulation) using a data acquisition controller (IDAC-4, Syntech, Kirchzarten, Germany) and analyzed with AUTOSPIKE v. 3.9 software (Syntech, Kirchzarten, Germany). The filter setting was 500 Hz at low cutoff and 3 kHz at high cutoff. Responses were calculated by counting the number of action potentials 1 s after stimulation.

### Phylogenetic Analysis

Sequences of *O. furnacalis* were based on the transcriptome data (Yang et al., 2015). Sequences from other *Ostrinia* species were from the reported references (Miura et al., 2009, 2010; Yasukochi et al., 2011) and downloaded through NCBI. The amino acid sequences of pheromone receptors were aligned by MAFFT^2^. Phylogenetic tree was constructed and analyzed by bootstrap test with 1000-resampling through RAxML version 8 with the Jones-Taylor-Thornton amino acid substitution model (JTT) (Stamatakis, 2014).

### Statistical Analysis

Data in the present study were normalized by log(X+1) and represented as mean ± SEM. The differences of responses to each pheromone components were analyzed by One-Way ANOVA and followed Duncan test (*P* < 0.05) by SPSS 20.0 (IBM, Endicott, NY, United States).

## Results

### Gene Cloning and Sequence Analysis of Pheromone Receptors in *O. furnacalis*

**FIGURE 1:**
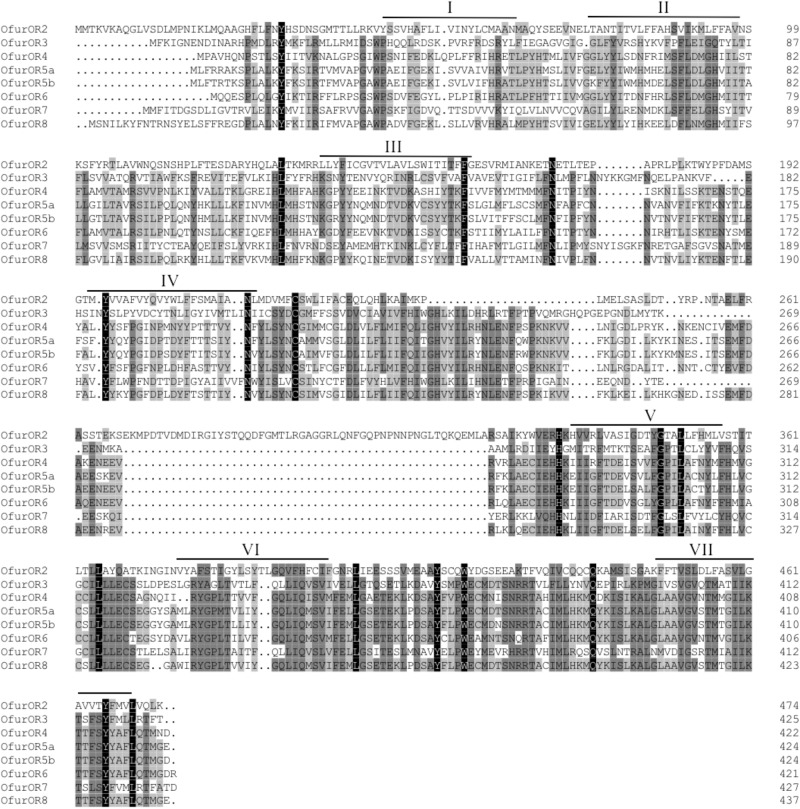
Alignments of amino acid sequence of odorant receptor co-receptor and pheromone receptors in *O. furnacalis*. Transmembrane domains were predicted by TMHMM Server Version 2.0 (http://www.cbs.dtu.dk/services/TMHMM/) and multiple alignments and identity calculation were done by the DNAMAN 6.0 (Lynnon Biosoft, United States). Predicted seven-transmembrane domains are marked with roman numbers. Amino acid numbering is given on the right of the alignment. Gaps in the alignment are indicated by a dash.

All the pheromone receptor names in this study were followed Yang et al. (2015). The naming system of pheromone receptors among *O. furnacalis*, *O. nubilalis*, and *O. scapulalis* were shown in **Table 1**. Full length of amino acid sequences of the pheromone receptors (ranged from 421 to 474aa) and the predicted seven transmembrane domains were shown in **Figure 1**. The identity between all pheromone receptors was 58.66%. Among all the pheromone receptors, OfurOR5a and OfurOR5b shared high similarity and their identity was 88.21%. Identities among other receptors were significantly lower (e.g., OfurOR8/Ofur5a, 71.30%; OfurOR4/OfurOR6, 64.71%; OfurOR1/OfurOR3, 64.08% etc.). OfurOR1 was not cloned from the strain we used.

**Table 1:** Name system of functionally characterized pheromone receptors in genus *Ostrinia* between different research articles.

Species	*O. furnacalis*	* O. nubilalis*(Z-type)	*O. scapulalis* (E-type)
PR groups	This study; Yang et al., 2015	Wanner et al., 2010; Leary et al., 2012	Miura et al., 2009, 2010

OR1	OfurOR1	OnubOR5	OscaOR1
OR3	OfurOR3	OnubOR4	OscaOR3
OR4	OfurOR4	OnubOR3	OscaOR4
OR5	OfurOR5a	OnubOR1	OscaOR5
	OfurOR5b		
OR6	OfurOR6	OnubOR6	OscaOR6
OR7	OfurOR7	–	OscaOR7
OR8	OfurOR8	–	OscaOR8

### OR4 and OR6 Are Main Receptors for Z/E12-14:OAc

**FIGURE 2:**
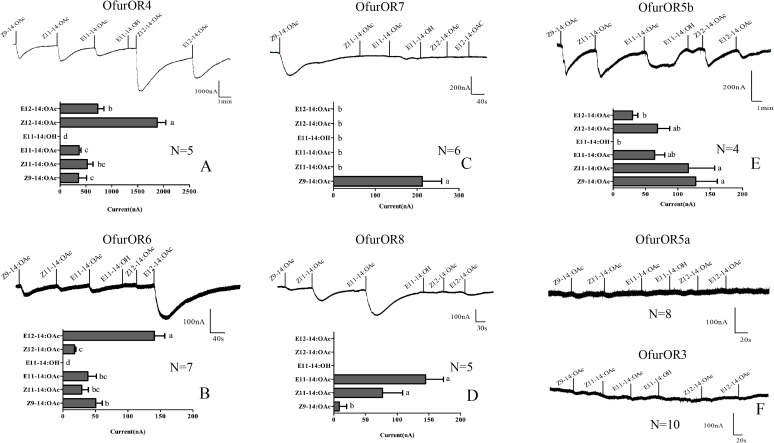
Representative current traces of OfurORn in response to pheromone components using *Xenopus* oocyte system. All the receptors were co-expressed with OfurOR2. Pheromone components (100 μM) were applied for 15 s at the time indicated by black line. Bar graph showed the statistical differences in response to each pheromone components (Mean ± SEM). **(A)** OfurOR4; **(B)** OfurOR6; **(C)** OfurOR7; **(D)** OfurOR8; **(E)** OfurOR5b; **(F)** OfurOR5a and OfurOR3. Bars labeled with different lowercase letters are significantly different.

OfurOR4 mainly responded to the main sex pheromones of *O. furnacalis*, Z12-14:OAc and E12-14:OAc, with the current values of 1876.8 ± 165 and 727.9 ± 120.4 nA, respectively. Both of the responses are significantly higher than that to other components (358.2 ± 156.6 nA < currents < 526.2 ± 110.1 nA, *F* = 31.821, *P* < 0.001, *N* = 5) (**Figure 2A**). OfurOR6 showed a much lower response to E12-14:OAc compared to OfurOR4, with the current value of 140.7 ± 6.0 nA, but the response was still significantly higher than that to other components (17.9 ± 2.2 nA < currents < 50.6 ± 10.0 nA, *F* = 33.490, *P* = 0.000, *N* = 7) (**Figure 2B**). Considering the effect for the applying order of the components, we used different order for OfurOR4, which E12-14:OAc was firstly applied to the oocytes, the response to E12-14:OAc became extremely strong (current > 3404.5 nA, *N* = 2) and even inhibited the response of Z9-14:OAc, Z11-14:OAc, and E11-14:OAc (**Supplementary Figure S1**).

### OR5b, OR7, and OR8 Broadly or Narrowly Tuned to Other Pheromones

OfurOR7 showed a specific response to one pheromone component Z9-14:OAc, with the current value of 212.2 ± 46.3 nA (*F* = 21.053, *P* = 0.000, *N* = 6) (**Figure 2C**). OfurOR8 significantly responded to Z/E11-14:OAc (*F* = 45.2210, *P* = 0.000, *N* = 5), with the current values of 76.8 ± 14.1 nA(Z) and 144.6 ± 28.3 nA(E), respectively (**Figure 2D**). Besides, a weak response to Z9-14:OAc (8.5 ± 5.2 nA) was also found in OfurOR8. Interestingly, OfurOR5a and OfurOR5b shared high sequence similarity, but only OfurOR5b responded to the pheromone components. OfurOR5b broadly tuned to Z9-14:OAc, E11-14:OAc, Z11-14:OAc, E12-14:OAc, and Z12-14:OAc. The responses to Z9-14:OAc, Z11-14:OAc were significantly higher than to E11-14:OAc, E12-14:OAc, and Z12-14:OAc (*F* = 4.155, *P* = 0.000, *N* = 4) (**Figure 2E**). OfurOR5a and OfurOR3 did not respond to any pheromone compounds supplied in this study (**Figure 2F**).

### Electrophysiological Analysis of the Male *s. trichoidea*

**FIGURE 3:**
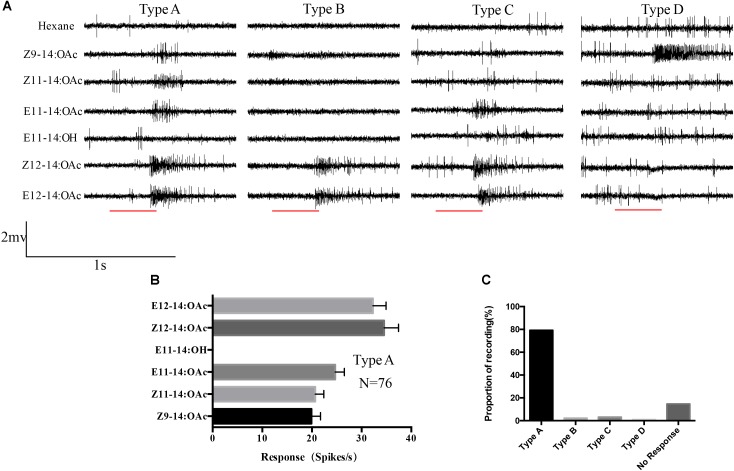
Single sensillum recordings of *s. trichoidea* from male adults in *O. furnacalis*. **(A)** Four different types (Type A–D) of *s. trichoidea* characterized by the response to pheromone components. The stimulus was applied for 300 ms which was represented with a red line under the trace. **(B)** Type A sensilla in response to pheromone components (10 μg). Data are represented as Mean ± SEM (*N* = 76). **(C)** The proportion of different sensilla type recorded in this study.

The single sensillum recordings were performed on the *s. trichoidea* of male antennae. In total 95 *s. trichoidea* were successfully recorded, among them, 82 sensilla responded to the provided pheromone components. Spontaneous activity often indicated more than one class of spike amplitudes that suggested that spikes from more than one neuron were recorded. But it was difficult to discriminate how many neurons in one sensillum or which neuron was responsible for the stimuli because the boundary between spikes was unclear. Four types (A–D) of sensilla were observed in which most of them were Type A (79.2%, 76/96) and they responded to all the provided pheromones except E11-14:OH. The mean responses to Z/E12-14:OAc were relatively higher than other components but no significant difference between them in Type A sensilla (**Figure 3**). Other types were also observed but the abundance was very low, with the number of 2 (Type B), 3(Type C), and 1(Type D). Type C sensilla responded to three components: E11-14:OAc, Z/E12-14:OAc. Type B and Type D showed specific response to Z/E12-14:OAc and Z9-14:OAc, respectively (**Figure 3**).

## Discussion

The genus *Ostrinia* has been treated as the model system to study sex pheromone communication because sex pheromone components have been identified in nine species and many species use same pheromone components with different proportion. We cloned seven sex pheromone receptors based on the previous transcriptomic study (Yang et al., 2015) and reviewed the names of the deorphanized pheromone receptor system in different *Ostrinia* research articles (**Table 1**). Unlike *Bombyx mori* (Sakurai et al., 2004; Nakagawa et al., 2005), in which the main pheromone receptors were narrowly tuned, most of pheromone receptors in *O. furnacalis* were broadly tuned to the pheromone components in *Xenopus* oocyte system. The result was basically consistent with the previous studies (Miura et al., 2009; Wanner et al., 2010; Leary et al., 2012). Among all pheromone receptors, OfurOR4 had significantly stronger response than the other tested receptors. The possible reason might be the system we used was heterologous expression system. When the pheromone receptor expressed *in vivo* there are other factors which affect the odor perception like OBPs, SNMP, etc. It was reported that the PBPs could increase the sensitivity of PRs to pheromones (Chang et al., 2015). Other receptors we tested might need OBP or SNMP to achieve higher sensitivity. It was also possible that other receptors need to expressed together to form a channel to achieve higher sensitivity coordinately. In *O. nubilalis*, different ORs could be observed in one neuron by *in situ* hybridization (Koutroumpa et al., 2014).

OfurOR4 has been identified as the receptor which could equally response to main components Z12-14:OAc and E12-14:OAc (Leary et al., 2012). Our results were basically consistent with the previous study. Z/E12-14:OAc might share same binding sites and could interfere with each other thus stimulate order could affect the results of the recording. That might cause the difference in response of the different stimuli order for OfurOR4. We found the additional receptor (OfurOR6) for main component E12-14:OAc. It seems that *O. furnacalis* need OfurOR4 and OfurOR6 to perceive its pheromone components coordinately, but the mechanism need to be further studied. *O. furnacals* use Z12-14:OAc and E12-14:OAc with ratio of 1:1 (Cheng et al., 1981; Huang et al., 1998b). In the field test, any trap lure loaded with a ratio other than 1:1 of Z/E12-14:OAc (more Z12 or E12) will cause the reduced captures (Cheng et al., 1982). Thus OfurOR4 might receive specific ratio of 1:1 Z/E components to initiate mating behavior. If the ratio deviates from 1:1 like more E12, OfurOR6 might have specific response to this redundant part of E12 and initiate antagonistic behavior together with OfurOR4.

It is interesting that the phenomenon of gene duplication for pheromone receptors in *Ostrinia* is very common. Various duplicates for pheromone receptors could be observed in each OR group (Yasukochi et al., 2011). In *O. furnacalis*, functional differentiation of gene duplication was found in OfurOR5. Similar phenomenon was found in other Lepidopterans. In *Helicoverpa armigera*, HarmOR14 and HarmOR14b shared high degree of identity but with different function *in vitro*. HarmOR14b responded to Z9-14:Ald whereas HarmOR14 did not response to any of *H. armigera* pheromone components (Liu et al., 2013; Chang et al., 2016). In *Agrotis segetum*, AsegOR1, AsegOR6-10 share high levels of amino acid sequence identity with each other (>70%), whereas their function were dramatically different (Zhang and Löfstedt, 2013).

OfurOR7 showed a specific response to Z9-14:OAc which is the sex pheromone component of *O. zaguliaevi* and *O. zealis* (Huang et al., 1998a; Ishikawa et al., 1999a) and a behavioral antagonist (Takanashi et al., 2006). OfurOR8 mainly responded to Z11-14:OAc and E11-14:OAc which were the sex pheromone components of *O. nubilalis* sex pheromone (Roelofs et al., 1985). Thus, OfurOR7 and OfurOR8 might be involved with interspecific recognition in *Ostrinia* species. Besides, OfurOR7 is the only one of pheromone receptors which highly expressed in male and female simultaneously (Yang et al., 2015), indicate that Z9-14:OAc might be an important pheromone clue for both sexes. Those receptors might contribute to reproductive isolation between *Ostrinia* species.

**FIGURE 4:**
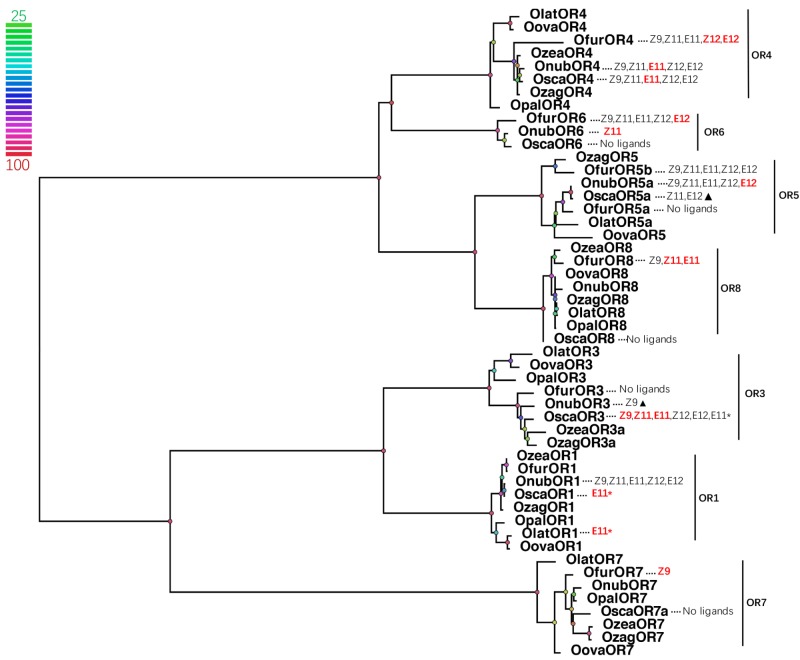
Phylogenetic tree of PRs and correspond *Ostrinia* pheromone ligands. Node color indicates the bootstrap values. (Bold and red letters indicated the components which the receptor mainly responded; Z9 = Z9-14:OAc, Z11 = Z11-14:OAc, E11 = E11-14:OAc, Z12 = Z12-14:OAc, E12 = E12-14:OAc, E11^∗^ = E11-14OH; pheromone receptors from *O. nubilalis* were renamed follow the system in **Table 1**, OnubOR1 = OnubOR5, OnubOR3 = OnubOR4, OnubOR4 = OnubOR3, OnubOR5a = OnubOR1, The former was the name we use for phylogenetic analysis, the later was the name reported from reference in **Table 1**; triangles represented the response was very weak).

Phylogenetic relationship showed that each OR group (OR1, OR3-8) in *Ostrinia* formed a clade and shared high degrees of identity (81.35–97.44%) (**Figure 4**). But most of the response pattern, especially for receptor responsible to the main pheromone components, was quite different among those closely related *Ostrinia* species when compare with previous studies. In genus *Ostrinia*, the ratio of the Z/E main pheromone components was usually considered to regulate the mating behavior. Those different response patterns make that mechanism more complex and need to be solved case by case. *O. nubilalis* and *O. scapulalis* used same pheromone components with same ratio (Z/E11-14:OAc, 97:3-Ztype, and 1:99-Etype) (Glover et al., 1987; Ishikawa et al., 1999b), OnubOR4 mainly responded to E11-14:OAc and OnubOR6 responded to Z11-14:OAc, the response values were equal in this two main receptors (Wanner et al., 2010). OscaOR4 showed a similar response compared to OnubOR4, but no response of OscaOR6 to any pheromones (Miura et al., 2010). *O. furnacalis* used Z/E12-14:OAc with 1:1 ratio which was quite different from *O. nubilalis* and *O. scapulalis*, OfurOR4 equally responded to Z12-14:OAc and E12-14:OAc, OfurOR6 mainly responded to E12-14:OAc different from OnubOR6 which responded to Z components. Comparisons of other pheromone receptors in *Ostrinia* were listed in **Figure 4** in detail.

The results of single sensillum recordings were basically similar to the previous studies (Takanashi et al., 2006; Domingue et al., 2007), most of the sensilla (Type A) responded to five pheromone components but we failed to distinguish the exact neurons. Corresponding to the results from *Xenopus* oocyte system, it seems that multiple the pheromone receptors were expressed on the neurons in Type A sensilla. We found three other types in which Type B sensilla only responded to Z/E12-14:OAc, indicated that the neuron in these sensilla might specifically express OfurOR4 and OfurOR6. Similarly, Type D of which specifically responded to Z9-14:OAc are possibly associated with the expression of OfurOR7. Type C responded to E11-14:OAc, Z/E12-14:OAc, which similar to Type A but difficult to speculate the expressed receptors in these sensilla according the results from *Xenopus* oocyte system. Possibly because the pheromone receptors co-expressed in *O. furnacalis*, that pattern has been reported in its closely related specie *O. nubilalis* (Koutroumpa et al., 2014). We did not find any neuron that responded E12-14:OH. In *Ostrinia*, OscaOR1, and OlatOR1 has been reported for responding E12-14:OH (Miura et al., 2009). Thus OfurOR1 might has same response profile. OfurOR1 could not be cloned in our strain and also not exist in the transcriptome (Yang et al., 2015) might indicated the degeneration of OfurOR1 in the colony we used. And it can also be that expression level of OfurOR1 is too low. Utilization of *in situ* hybridization and CRISPR-Cas9 might further elucidate the neuron distribution and receptor expression pattern in single sensillum.

## Author Contributions

WL, BY, and G-rW designed the research, analyzed the data, and wrote the paper. WL and SC performed the research. X-cJ provided biological samples.

## Conflict of Interest Statement

The authors declare that the research was conducted in the absence of any commercial or financial relationships that could be construed as a potential conflict of interest.

## Footnotes

1 http://www.cbs.dtu.dk/services/TMHMM/

2 https://www.ebi.ac.uk/Tools/msa/mafft

## Supplementary Material

FIGURE S1 Current traces of OfurOR4/OfurOR2 in response to pheromone compounds (100 μM) with different order.

TABLE S1 Primers of pheromone receptors used for PCR.
