# Supplementary figures and images for: Functional Studies of Sex Pheromone Receptors in Asian Corn Borer Ostrinia furnacalis
Source: Front Physiol. 2018 May 23;9:591. doi: 10.3389/fphys.2018.00591 (PMC5974041; doi:10.3389/fphys.2018.00591)

E12-14:OAc

Z12-14:OAc

Z9-14:OAc

Z11-14:OAc

E11-14:OAc

E11-14:OH

2000nA

2min

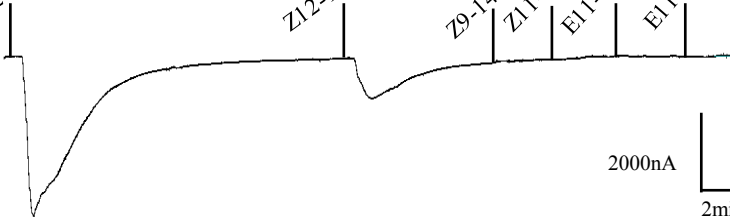

Supplement: FIGURE S1 — Current traces of OfurOR4/OfurOR2 in response to pheromone compounds (100 μM) with different order. [file Image_1.PDF]
