# Supplementary material for: Functional Studies of Sex Pheromone Receptors in Asian Corn Borer Ostrinia furnacalis
Source: Front Physiol. 2018 May 23;9:591. doi: 10.3389/fphys.2018.00591 (PMC5974041; doi:10.3389/fphys.2018.00591)
Supplement: TABLE S1 — Primers of pheromone receptors used for PCR. [file Table_1.DOCX]

Table S1. Primers of pheromone receptors used for PCR.

| Genes | Primers-F | Primers-R |
| --- | --- | --- |
| *OfurOR2* | ATGATGACCAAAGTGAAAGCTC | CTACTTCAGTTGTACCAAAACCATG |
| *OfurOR3* | ATGTTTAAGATTGGAAATGAAAAC | TTAGGTGAATGTTCGCAGTAGC |
| *OfurOR4* | ATGCCCGCCGTTCACC | TTAATCATTCATTGTTTGTAGGAATG |
| *OfurOR5a* | ATGTTATTCAGAAGGGCAAAAAG | CTACTCTCCCATTGTTTGCAGA |
| *OfurOR5b* | ATGTTATTCACAAGAACAAAAAGTCC | CTAATCTCCCATTGTTTGCAGA |
| *OfurOR6* | ATGCAACAGGAATCGCCA | TTATCTATCTCCCATTGTTTGTAAA |
| *OfurOR7* | ATGATGTTCATCACTGATGGAAGTG | TTAATCATCAGTAGCAAATGTCCG |
| *OfurOR8* | ATGAGCAACATTTTAAAATATTTTAAC | CTACTCTCCCATTGTTTGCAG |
